# Adaptation and testing of psychosocial assessment instruments for cross-cultural use: an example from the Thailand Burma border

**DOI:** 10.1186/s40359-014-0031-6

**Published:** 2014-08-31

**Authors:** Emily E Haroz, Judith K Bass, Catherine Lee, Laura K Murray, Courtland Robinson, Paul Bolton

**Affiliations:** Department of Mental Health, Johns Hopkins Bloomberg School of Public Health, 624 N. Broadway, Room 780, Baltimore, MD 21205 USA; Department of International Health, Johns Hopkins Bloomberg School of Public Health, Baltimore, MD USA

**Keywords:** Validation, Refugee, Psychometrics, Instrument development

## Abstract

**Background:**

The purpose of this study was to develop valid and reliable instruments to assess priority psychosocial problems and functioning among adult survivors of systematic violence from Burma living in Thailand.

**Methods:**

The process involved four steps: 1) instrument drafting and piloting; 2) reliability and validity testing; 3) instrument revision; and 4) retesting revised instrument.

**Results:**

A total of *N* = 158 interviews were completed. Overall subscales showed good internal consistency (0.73-0.92) and satisfactory combined test-retest/inter rater reliability (0.63-0.84). Criterion validity, was not demonstrated for any scale. The alcohol and functioning scales underperformed and were revised (step 3) and retested (step 4). Upon retesting, the function scale showed good internal consistency reliability (0.91-0.92), and the alcohol scale showed acceptable internal consistency (0.79) and strong test-retest/inter-rater reliability (0.86-0.89).

**Conclusions:**

This paper describes the importance and process of adaptation and testing, illustrated by the experiences and results for selected instruments in this population.

## Background

It is estimated that up to fifty percent of displaced persons worldwide present with mental health problems (World Health Organization (WHO) 2013). De Jong et al. (2003), in their review of mental health disorders in areas of conflict and displacement, found that these populations are at an increased risk for depression, anxiety and PTSD-like symptomology. This increased risk level is especially true for individuals directly exposed to violence. Much of the research on mental health issues among displaced populations has been conducted in higher resource countries with resettled populations. Less is known about displaced persons located in countries with few health and mental health services (Bass et al. 2007).

Local testing of the reliability and validity of psychological measures in non-western settings is an ongoing challenge, particularly in low-resource contexts. While there is agreement that instruments developed in western-based populations cannot simply be translated and back-translated, there is a lack of agreement on standards in adaptation and validation of instruments, including disorder screeners and scales (Kohrt et al. 2011). Without prior testing of the appropriateness of these measures, the accuracy of study conclusions that use them is unknown. Unfortunately, validation of assessment instruments is not the common practice in global mental health.

In the current paper we describe the development and testing of multiple instruments to assess psychosocial problems and functioning among adult survivors of systematic violence from Burma, currently living in Mae Sot, Thailand. Only two studies have systematically looked at the mental health of Burmese refugees living in Thailand (Allden et al. 1996; Cardozo et al. 2004). Respectively, these studies found elevated symptom levels for depression and PTSD among young-adult Burmese in Bangkok and Karenni refugees in displaced persons camps in Thailand. Both studies used self-report measures – the Harvard Trauma Questionnaire (Mollica et al. 1987) and the Hopkins Symptom Checklist-25 Items (Winokur et al. 1984; Hesbacher 1980) - previously tested and validated in international and resource limited settings (Silove et al. 2007; Betancourt et al. 2009; Jakobsen et al. 2011) but not among the current study population.

The aim of this study was to develop a set of reliable and valid instruments that could be used as screening, monitoring and evaluation tools in a subsequent Randomized Controlled Trial (RCT) of a psychotherapeutic intervention. The study consisted of the following steps: 1) instrument drafting including pilot testing, 2) reliability and validity testing, 3) revision based on results from step 2, and 4) re-testing of revised measures. This four-step process was part of a larger field-based methodology to inform the design, implementation, monitoring and evaluation of community-based services to address the mental health problems of this population (Applied Mental Health Research Group 2013, module 1).

## Methods

### Step 1: Instrument adaptation

A prior qualitative study of local perspective on problems of Burmese migrants and displaced persons living outside of refugee camps in Thailand identified two main groups of symptoms related to depression and trauma (Lee et al. 2011). A review of existing instruments suggested that the Hopkins Symptom Checklist 25-item version (HSCL) (Winokur et al. 1984; Hesbacher 1980), which includes a depression and an anxiety sub-scale, and the Harvard Trauma Questionnaire (HTQ) (Mollica et al. 1987) were appropriate instruments for adaptation based on how closely they tracked with the local problem descriptions. An alcohol measure, the Alcohol Use and Disorders Identification Test (AUDIT (Saunders et al. 1993) was also included and adapted to reflect the problems with alcohol that were apparent from the qualitative study. Adaptation included translation based on local idioms and phrases from the qualitative study, and addition of items specifically relevant to the local context, also from the qualitative study. Both the depression and anxiety scales included 2 additional items described in the qualitative study, but not found in the standard versions. No additional items were added to the trauma symptom scale (HTQ) or the alcohol use scale (AUDIT). This adaptation process has been documented more fully elsewhere (Bass et al. 2008; Bolton et al. 2004) and in a detailed manual describing this approach (Applied Mental Health Research Group 2013, module 2).

An assessment of functionality was developed based on previously described methods (Applied Mental Health Research Group 2013, module 2; Bolton & Tang 2002). This aimed to measure the degree of difficulty people experienced when performing activities of daily living. These activities were derived from the qualitative study in which interviewees were asked to describe important tasks and activities men and women regularly perform to care for themselves, their families and their communities. Separate instruments were created for men and women reflecting gender-specific responses.

For the measures related to mental health problems, respondents were asked to report how frequently they experienced each symptom in the prior two weeks: “none of the time” (0), “a little of the time” (1), “some of the time” (2), “most of the time” (3) or “almost all of the time” (4). For the alcohol use scale, responses were based on how often a respondent experienced a certain type of drinking related experience: “never” (0), “monthly or less” (1), “2-4 times a month” (2), “2-3 times a week” (3) and “4 or more times a week” (4). For the function scale, respondents were asked how much difficulty they had with each activity in the prior two weeks: “no difficulty at all” (0), “a little bit” (1), “a moderate amount” (2), “a lot” (3), “often cannot do” (4). If the respondent reported that a specific activity was not relevant to them (such as a woman without children being asked about caring for children) that activity was reported as not applicable and scoring was based on the remaining items.

An experienced Burmese translator translated the draft instrument using vocabulary from the qualitative interviews. Where a concept in the instrument was also represented in the qualitative data, the translator used the term from the qualitative study (22 out of the 37 symptoms), otherwise the translator used personal knowledge of local ways of talking about mental health problems. For the function questions and other questions directly derived from the qualitative study, the language for key terms was taken from the qualitative data. Bilingual English-Burmese staff affiliated with the project reviewed the resulting translation. The draft instrument was then back translated to english prior to interview training. Further adaptation, translation and clarification were done during the interviewer training with the local Burmese-speaking team and based on their input. At this final stage, changes to the instrument were made only when the change was not to terms derived from the qualitative data, the majority of the interviewers agreed that a change was needed, and they agreed on what the change should be.

The resulting set of instruments is referred to here as the Mental Health Assessment Project Instrument (MHAP-I). The MHAP-I included the following sections: traumatic experiences, 25 items, measured using HTQ (e.g. experienced or witnessed “detention”; “forced labor”); Posttraumatic stress symptoms, 30 items, measured using the HTQ (e.g. “In the last month, how often have you experienced feeling as though the event is happening again?”); depression symptoms, 17 items, measured using the HSCL and two additional local items (e.g. “In the last month, how often have you experienced hopeless; don’t care what will happen?”; “In the last month, how often have you experienced disappointment?”); anxiety symptoms, 10 items, measured using the HSCL and two additional local items (e.g. “In the last month, how often have you experienced heart beating quickly?”); alcohol use, 10 items, measured using the AUDIT (e.g. “How often do you have a drink containing alcohol?”); and functional impairment, 11 items for men and 14 items for women derived from previous qualitative work (e.g. “in the last month, are you having no more difficulty than most other men/women of your age, a little more, a moderate amount, a lot more, or you often cannot do this task: farming?”).

A pilot study explored the interview procedure and the MHAP-I questions for both interviewers and respondents. Interviewers administered the MHAP-I to 18 adults (9 men; 9 women) from the target population. At the end of each interview, the respondents were asked to report what they liked and did not like about the interview as well as whether there were any questions they found difficult to understand or answer. The study team reviewed the results in order to further refine the MHAP-I.

### Step 2: Reliability and validity testing

#### Sample recruitment

The sample for this study was recruited through consultations with Key Informants (KIs). KIs from each of the three local partner organizations were identified during the previous qualitative study by participants from the free listing exercise and by the leadership of the local partner organizations. KI’s were said to be particularly knowledgeable about the mental health problems that arose from the free listing exercise by the participants in the free-listing exercise and the leadership of the local partner organizations. The KIs were members of the study population (i.e. not considered outsiders), and included a former political prisoner who was a member of AAPP, a staff member of SAW who oversaw the running of several safe houses and boarding houses for women and children, and a mental health counselor from a local medical clinic. None of the KIs had extensive clinical training, but all were members of the displaced Burmese community, had worked in human services within the displaced Burmese community for a number of years, and were particularly knowledgeable about local perceptions of mental health problems.

Prior to recruiting the sample, KIs were provided with a brief information sheet that included problems that arose during the free listing and which corresponded to signs and symptoms of depression and PTS (e.g. disappointment, trouble making decisions, problems with sleep, bad dreams, flashbacks, distressing memories). KIs were then asked to think about people they knew who currently have or do not have many (not necessarily all) of the signs and symptoms. KIs developed three lists based on their organization’s client population who were in the given age group and who they knew well: 1) persons the KIs were confident had depression symptoms, 2) persons they were confident had trauma symptoms, and 3) persons they were confident did not have either depression or trauma symptoms.

The KIs contacted each individual on their lists and confidentially asked if the person felt he/she had either of these mental health problems, both problems, or neither problems and whether he/she would be willing to be contacted again to be interviewed for a study. Specifically, respondents were asked whether they had symptoms associated with “Sait Dat Cha Mu” (depression), “Sai Ka Ya Pyit Bi Naut Pyit Baw Thaw Kyaw Ah Nay” (trauma), or “Sait Kyamayae Pyit Tha Na Ma Shi” (neither depression nor trauma). Prior to contacting the respondents, KIs were instructed on how to ask individual’s opinions in non-leading ways. When the KIs contacted the respondents they explained that the project was working to create a survey for the community to provide assistance to those in need and participants were needed to help test the survey. KIs also explained to respondents that the information they provide would be kept confidential and private. KIs were instructed to record the exact response the individual gave. Those respondents whose self-report agreed with the KIs’ opinion were retained in the sample; while those respondents whose self-report disagreed with the KIs’ opinion were not included in the study.

This procedure was done to determine case/non-case status. For example, if the respondent self-reported having depression symptoms and the KI independently reported that respondent was depressed, then the respondent would be considered a case. Similarly, if a KI identified a respondent as not having symptoms related to depression and/or trauma and the respondent self-reported no symptoms related to depression and/or, then this person would be classified as a non-case.

The procedure allowed for overlap between the depression and trauma symptom lists, as respondents could be classified as either having only depression, only trauma symptoms, or having both. The final sample consisted of people classified as cases (experiencing depression and/or trauma-related symptoms) and non-cases (not experiencing symptoms related to depression and/or trauma). This process of local case-identification is described in detail elsewhere (Applied Mental Health Research Group 2013, module 2) and is a method that has been successfully used in some validation studies in low-resource contexts (Bass et al. 2008; Bolton 2001) while it has been less successful in others. Success is dependent on finding informants who are knowledgeable about the mental health of the participants.

All participants provided informed verbal consent for their participation in the study and all study procedures were approved by the Johns Hopkins Bloomberg School of Public Health Internal Review Board (IRB; #3348) and a local ethics committee in Mae Sot, Thailand. The local ethics committee included five members, all Burmese, from local non-government and community-based organizations. All members were knowledgeable about local mental health and human rights issues affecting the displaced population. The local committee reviewed translated copies of all study documents and procedures and provided written approval for the trail.

#### Analysis

Testing reliability and validity was based on syndrome and function scales within the MHAP-I. Scores for the depression and trauma syndrome scales and function (male and female) scale represented the mean response across all items in that scale except for the alcohol scale, which was scored as the sum of the item responses. All missing item scores were imputed using single mean imputation. Reliability and validity testing of the MHAP-I subscales included evaluation of combined test-retest/inter-rater reliability, internal consistency reliability, and criterion validity. All analyses were done using STATA Statistical Software StataCorp 2009.

#### Reliability

Evaluation of combined test-retest/inter-rater reliability was done by re-interview using the MHAP-I within 4 days of the original administration. The re-interview was done by an independent and different interviewer. All interviewees were asked if they would be willing to be interviewed a second time. Of those interviewees who agreed to be contacted again, 20% were randomly selected for re-interview with the same instrument within four days. A total of *n =* 31 participants were re-interviewed. Test-retest/inter-rater reliabilities were assessed using a Pearson correlation coefficient (*r*), which provides a measure of how similar scale scores are on the initial interview compared with the re-interview assessment. For all scales, scatterplots suggested a linear relationship between first and second interview scores, a requirement for use of the Pearson correlation coefficient. Internal consistency reliability was evaluated by calculating Cronbach’s Alpha (α) and item level analysis examining item-scale correlation for each scale (with the exception of the HTQ experiences scale).

#### Validity

Criterion validity was only assessed for the depression symptom and trauma symptom scales as these were the main screening criteria and outcomes for the RCT. To measure criterion validity, the study relied on the list of cases and non-cases. All individuals on the lists were assigned an identification number known only to the study coordinators in order to blind interviewers to the classification as case/non-case and to maintain confidentiality of study forms. Local-criterion related validity was investigated by comparing average symptom scores across case/non-case groups and would be supported if cases of depression/trauma had statistically and clinically significantly higher mean scale scores on the depression and trauma scales compared with non-cases (those with no mental health problems).

### Steps 3 & 4: Instrument revision and retesting

Based on the poor reliability results for the function and alcohol use measures (step 2, results presented below), further revision and retesting were necessary. For the function measure, additional qualitative data was gathered using focus groups to inform these revisions. Many respondents answered “not applicable” (most frequently to “farming” and “take kids to school; pick up kids”) so more information was needed to explore whether the activities in the function measure did, in fact, fit with individual’s actual daily tasks.

Focus group discussions were held with 4 groups of women (*n* = 4, *n* = 4, *n* = 3, *n* = 9), 2 groups of men (*n* = 3, *n* = 10), and one gender-mixed group (*n* = 5). Participants were selected based on their professional work with, and knowledge of, the study population in the local area and included interviewers from step 2 and representatives from the local partner organizations. Each focus group generated two lists: 1) tasks and activities that most men/women need to do to care for themselves, family, and community that people may currently have difficulty performing and 2) tasks and activities that people who feel a lot of emotions or have something like depression or sadness typically have difficulty performing. The second list was an addition to the approach used in the initial qualitative study that was used to identify activities more likely to be impacted by mental health conditions and thus by treatments. Each focus group generated lists separately for men and women. The lists generated by the first male and female focus groups were presented to subsequent groups who were asked if they agreed or disagreed with the lists. Only items that were agreed upon by all seven focus groups were included and combined with the well-performing original items for the revised version of the function scale (Table 1).

**Table 1:** **Items on the final version of the MHAP-I function scale**

**Q. In the last month, how much difficulty have you had** ***ACTIVITY*** **, compared to other men/women your age…**		
	**Women**	**Men**
1	Watching TV^a^	Watching TV^a,b^
2	Doing cooking^a,b^	Accessing information about the things you are interested in^b^
3	Going shopping, buying food^a^	Walking around the area^b^
4	Accessing information about the things you are interested in^b^	Doing work for income^a^
5	To wash/iron clothes^a,b^	Doing cooking^a^
6	Walking around the area^b^	Repairing your house^b^
7	Socializing with friends^a,b^	Doing religious activities^a,b^
8	Doing religious activities^a,b^	Playing sports^a,b^
9	Doing housework (in your home)^a,b^	Socializing with friends^a,b^
10	Doing work for income^a^	Going to the market^b^
11	Taking your kids or children for whom you are responsible to school and picking up from school^a,b^	Making new friends^c^
12	Look after your kids or children from whom you are responsible^a^	Keeping yourself maintained well, such as dressing well or shaving and grooming^a^
13	Eating together with others^c^	Planning for and preparing for the next day’s activities^b^
14	Communicating with others^c^	Eating together with others^c^
15	Managing money for the household	Going out^a^
16	Responding to changes in the daily schedule that come up or dealing with problems that come up which are out of the regular routine^c^	Spending time with family and friends^b^
17	Giving encouragement to friends when they need support^b^	
18	Playing with your kids or children for whom you are responsible^b^	
19	Planning for and preparing for the next day’s activities^b^	
20	Grooming yourself such as styling your hair or dressing well^a,b^	
21	Tidying up things in your house versus just doing the minimum cooking, washing and basic cleaning^c^	
22	Making new friends^c^	
23	Spending time with family and friends^b^	

^a^Items from original version of function scale (these items reflect final wording after revision).

^b^Items added during revision: tasks and activities that are difficult for people in the general community.

^c^Items added during revision: tasks and activities that are difficult for people who are feeling a lot of emotions.

For the alcohol use scale (AUDIT), we reviewed and found problems with the clarity and meaning of the translation. For example, one question read "Have you or someone else been injured because of drinking?" whereas the original wording was “Have you or someone else been injured as a result of your (emphasis added) drinking?” The problematic questions were revised. A visual response aid was also added that included photos of common local drinks (brands of local beer and whiskey) and the amount of each substance that was considered a standard drink.

The revised function and AUDIT scales were retested among a small convenience sample of people from Burma living in the study area who were thought to have similar problems to the target population in order to evaluate combined test-retest/inter-rater and internal consistency reliability. Individuals were administered the revised scales by a trained interviewer and a second interviewer followed-up with the respondent 2–5 days later.

## Results

### Results from steps 1 & 2

The pilot study found that the MHAP-I and interview process were acceptable and understandable to the interviewees. Out of the total of *n =* 222 names on the case/non-case lists; 205 (92% agreement between KI and individual) were classified as concordant (i.e. the respondent and KI agreed to their mental health status). Of this concordant sample, *n =* 158 (77%) people were interviewed. The 47 people not interviewed either refused or could not be located. Fifty-two percent (*n = 82*) of the sample were men. Ages of the respondents ranged from 18 to over 60 years, with most between 25 and 45. The range and distribution of demographic categories are presented in Table 2.

**Table 2:** **Study sample characteristics**

	**Step 2 N, (%)**	**Step 4 N, (%)**
**Total N**	158	66
**Gender**		
Male	82, (52%)	35, (53%)
Female	76, (48%)	31, (47%)
**Ages**		
18-24	29, (18%)	14, (21%)
25-34	58, (37%)	25, (38%)
35-44	43, (27%)	19, (19%)
45-59	24, (15%)	6, (9%)
>60	4, (3%)	0, (0%)^a^
**Marital status**		
Divorced	12, (8%)	9, (14%)
Married	88, (56%)	21, (32%)
Single	49, (31%)	26, (39%)
Widowed	9, (5%)	4, (6%)^b^
**Education**		
None	10, (6%)	6, (9%)
Primary school (1–4 standard)	13, (8%)	3, (5%)
Middle school (5–6 standard)	31, (20%)	17, (36%)
High school (9–10 standard)	28, (18%)	14, (21%)
More than high school (post 10)	76, (48%)	26, (40%)
**Primary ethnic group**		
Sgaw Karen	8, (5%)	11, (17%)
Pwo Karen	8, (5%)	0, (0%)
Shan	3, (2%)	1, (2%)
Pa-O	4, (3%)	0, (0%)
Mon	4, (3%)	2, (3%)
Burman	114, (72%)	43, (65%)
Other	17, (11%)	9, (13%)

^a^2 people missing age.

^b^6 people missing marital status.

Table 3 presents the reliability results. The depression, anxiety, and trauma symptom had acceptable combined test-retest/inter-rater reliability as evidenced by test-retest reliability of *r =* 0.84; *r =* 0.71; and *r =* 0.78 respectively. However, the HTQ experiences scale, the functioning scales, and the AUDIT did not. A sensitivity analysis examining whether any interviewers in particular were associated with lower agreement did not identify any significant variation by interviewer.

**Table 3:** **Steps 2 & 4 reliability results**

		**Step 2** ***N =*** **31)**		**Step 4 N =**		
**Test retest/inter rater reliability**	**Mean (** ***SD*** **) first interview**	**Mean (** ***SD*** **) repeat interview**	**Correlation (** ***r)***	**Mean (** ***SD*** **) first interview**	**Mean (** ***SD*** **) repeat interview**	**Correlation (** ***r)***
**Symptom scales**						
HSCL depression section score	15.77 (11.35)	11.74 (10.09)	0.84*	---------	---------	---------
HSCL anxiety section score	8.71 (7.11)	6.35 (5.08)	0.71*	---------	---------	---------
HTQ Symptom section score	21.97 (15.85)	14.45 (11.66)	0.78*	---------	---------	---------
**Function scales**						
Male sub scale	0.43 (0.52)	0.28 (0.3)	0.34	0.42 (0.59)	0.43 (0.60)	0.89*
Female sub scale	0.33 (0.38)	0.31 (0.25)	0.43	0.33 (0.54)	0.35 (0.54)	0.86*
**Alcohol scale**	2.13 (2.33)	2.68 (3.03)	0.63*	4.79 (6.20)	0.46 (5.88)	0.86*
**Internal consistency reliability**	**Total sample** **(** ***N*** ** = 158)**	**Males** **(** ***N*** ** = 82)**	**Females** **(** ***N*** ** = 76)**	**Total sample** **(** ***N*** ** = 66)**	**Males** **(** ***N*** ** = 35)**	**Females** **(** ***N*** ** = 31)**
**Symptom scales**						
HSCL depression section score	0.92	0.84	0.93	---------	---------	---------
HSCL anxiety section score	0.89	0.84	0.91	---------	---------	---------
HTQ Symptom section score	0.92	0.86	0.94	---------	---------	---------
**Function scale**	---------	0.83	0.83	---------	0.91	0.92
**Alcohol scale**	0.73	0.73	0.64	0.79	0.70	0.57

*Significant at *p* < 0.05.

Cronbach’s alpha scores are presented (Table 3) for each scale (except the HTQ experiences scale) for males, females, and the total sample. Alpha scores for all scales, except the AUDIT, were very good, as evidenced by scores greater than *α =* 0.70. The item analysis supported the removal of only one item, “Headache”, from the HSCL Anxiety Scale. Questions from the qualitative studies that were added to the various scales performed well, with correlations to the total scale as high as, or higher than, most of the standard items.

Table 4 describes the results of the local-criterion validity testing of the depression and trauma related symptom scales. These include the versions of the scales that include both standard items and the locally generated symptoms. Comparing total scale scores across case status, there were no statistically significant differences between ‘cases’ and ‘non-cases.’

**Table 4:** **Step 2 Criterion Validity**

	**Total sample (N = 158)**				**Total males (N = 82)**			**Total females (N = 76)**		
	**Score range max-min**	**Cases** ^**a**^ **(median)**	**Non-cases** ^**a**^ **(median)**	**Difference (p-value)** ^**b**^	**Cases** ^**a**^ **(median)**	**Non-cases** ^**a**^ **(median)**	**Difference (p-value)** ^**b**^	**Cases** ^**a**^ **(median)**	**Non-cases** ^**a**^ **(median)**	**Difference (p-value)** ^**b**^
All depression symptoms score (median)	0-62	12	14	−2 (0.41)	10.5	14	−3.5 (0.36)	17	15	2 (0.87)
All trauma symptoms score, median	0-93	16	16	0 (0.47)	14.5	16	−1.5 (0.3)	19	17	2 (0.93)

^a^‘Cases’ refers to participants who were said by a key informant familiar with their history to have the problem. ‘Non-Cases’ refers to survivors who were said by such a key informant to NOT have the problem.

^b^ *p value* for the statistical significance of the difference in scale scores by caseness, based on mean comparison T-tests.

### Results from steps 3 & 4

The original versions of the function scales and AUDIT were found to be unacceptable due to poor test-retest/inter-rater reliability, many N/A responses for the function scales, low internal consistency reliability and some problems with translation for the AUDIT. After revision, these scales underwent repeat testing (step 4). The sample for this repeat testing consisted of *n =* 66 individuals (men: *n = 35*; women: *n = 31*) with an average age of 34.5 years old (Table 2). The revised function index and the revised alcohol use scale showed good test-retest/inter-rater reliability (male functioning: *r* = 0.89, female functioning: *r* = 0.86; AUDIT: *r* = 0.86) and good internal consistency reliability (male functioning: α = 0.91, female functioning: α = 0.92; AUDIT: α = 0.79) (Table 3).

## Discussion

This paper describes the basic psychometric testing of a set of mental health screening measures among Burmese adult migrants and displaced persons living in Mae Sot, Thailand. We found that a standard scale for alcohol use (AUDIT) initially performed poorly, with only acceptable correlation for combined test-retest/inter-rater reliability and low internal consistency reliability. Review and adjustments led to significant improvements in the scale’s performance. Similarly, a locally developed function scale performed poorly on first use for the combined test-retest/inter-rater reliability and many participants responded with N/A on some questions (“farming” and “take kids to school; pick up from school”), suggesting that these activities were not as common as expected. This required a new qualitative study to generate new data to review and revise the instruments. The revised instrument showed marked improvement in combined test-retest/inter-rater and internal consistency reliability.

Standard symptom instruments found to be useful elsewhere – the HSCL and HTQ - also showed good combined inter-rater/test-retest reliability among this study population. Internal consistency reliability was also good and item analysis supported the removal of only one item. The addition of locally relevant items based on a prior qualitative study added to the breadth of the instruments and these items performed as well as standard items, but were few in number (2 items for depression and 2 items for anxiety) and did not appreciably affect testing results.

We did not demonstrate criterion validity, as evidenced by the lack of significant differences for depression and trauma scores between cases and non-cases (Table 4). The testing procedure depended on the existence and accurate identification of local people with and without the problems being measured (in this case depression and trauma-related problems) by informants who knew the participants well enough to express an informed opinion on the presence or absence of these problems. The lack of criterion validity we found for both the HSCL and the HTQ means either that both instruments could not discriminate between true cases and non-cases in this population or that it was the informants who were unable to do so.

It may also be possible that the instrument itself was problematic because adaptation relied on local lay understandings of mental health problems rather than professional sources of knowledge (e.g. local mental health professionals). If local mental health professionals are available in a location, it is important to include them in both the adaptation and testing of instruments. However, no such professionals existed in this community and instead we relied on local lay people (KIs) who were thought to be the most knowledgeable about mental health problems in the community and, to be available for the project. The KIs that were involved in adaptation of the MHAP-I (from both the previous qualitative research and different KIs involved during the interviewer training) were identified by community members interviewed during the free listing activities (see Lee et al. 2011) and by the local partner organizations. At the time, these KIs were thought to be the most knowledgeable people about mental health issues in this area. As such, the MHAP-I may only include signs and symptoms of depression and posttraumatic stress that are relevant to community members.

On further investigation related to the failed criterion validity, the cause did not appear to be local difficulty in recognizing these syndromes since the qualitative study found that people in Mae Sot understand depression and trauma related stress and believe that they are important issues affecting adults from Burma. Post study discussions with local partner organizations indicated that the KI’s felt that the problem might have been with the initial list making process; that despite having directions and knowing the people well, they may not have known enough about their emotional and personal situations to evaluate them. However, given the high concordance rate between the KI and the respondent (92%), the evidence does not support this conclusion. Another possible explanation was the possibility that the KI frequently influenced self-reports of mental health status, despite the efforts taken to ensure this didn’t happen. This type of bias is possible since, unlike previous studies, the KI who designated people as cases and non-cases was the same person collecting the self-reports and therefore was clearly not blinded to their assessment. If this is the cause then the KIs must have both frequently been incorrect in their assessments and frequently influenced respondents’ answers.

In previous studies measures have tended to either perform well across all measures of reliability and validity (including criterion validity testing) or poorly across all measures. In this case, the good performance of the HSCL and HTQ on the reliability tests, the concerns of the KIs as to their ability to discriminate between cases and non-cases, and the lack of blinding in the collecting of self-reports suggest that a failure in the criterion validity testing process is more likely than a failure with the instrument as a whole or the interviewers conducting the testing. The authors should have more clearly established that KIs felt able to confidently make these assessments before proceeding with this approach, and conducted the self-assessments blinded.

Other studies have also used ethnographic approaches to develop locally valid questionnaires (Betancourt et al. 2009; Bass et al. 2008; Miller et al. 2006). However assessing criterion validity in the absence of a gold standard remains a challenge, especially in non-western and low-resource settings. While we used a method for establishing criterion validity based on local opinion in this study, others have taken alternative approaches. Some studies have compared locally derived measures to standard measures used in the West, which are not locally validated (Rasmussen et al. 2011; Ertl et al. 2010). Other studies have relied on either local or foreign mental health professionals to perform diagnostic evaluations (Silove et al. 2007) as the criterion. Khort et al. (2011) propose the use of task-shifting, using non-psychiatrists’ evaluations (specifically psychosocial counselors) conducted by structured interviews related to psychosocial functioning as a criterion. Regardless of the method, where disagreement between these standards and the instrument suggests lack of criterion validity (e.g. local opinion, western measures, or psychiatrists or psychosocial worker diagnosis), a problem arises with deciding whether measure is accurate. There is a continued lack of true gold standards as a point of comparison.

A limitation of this study is that it relied on members of the community to inform adaptation and testing and as such the success of the methods described in this paper vary according to whether the concepts exist locally and how well local people can recognize them in themselves and those around them. Engaging with local mental health professionals would likely obviate this limitation. However, we continue to use this approach in situations, such as the current study, where no such mental health professionals existed in this area. This study was also limited by security concerns at the study location, due mainly to the fears of a largely undocumented migrant population to arrest and/or harassment by local authorities when traveling in and around Mae Sot. The study sample only included people in the community who had existing contact with one of the partner organizations and lived in certain neighborhoods in Mae Sot. Thus, the study was unable to sample people from outside these areas, who might have been more or less affected by these mental health problems.

## Conclusions

After local adaptation and translation, the depression and trauma symptom scales proved to be acceptable and understandable to the Burmese refugee population and performed well psychometrically with the exception of establishing criterion validity. The AUDIT scale and the locally developed function scale did not perform well at first and required revision and further testing before they were deemed acceptable.

By testing, revising, and retesting we were able to identify and correct problems that might have gone unnoticed and subsequently lead to low-quality data and incorrect conclusions in future studies. This process illustrates the importance of testing psychosocial instruments prior to use in clinical or epidemiological studies concerning the measurement of mental health symptoms. The criterion validity results indicate that we have yet to perfect the methodology of adapting psychometric scales in lower resource settings where there is a lack of an accepted gold standard.
